# Outcome of Femoral Angioplasty/Stenting Procedures in Different Ethnic Groups in England: A Retrospective Analysis of Hospital Episode Statistics and Review of Literature

**DOI:** 10.1177/15266028211070967

**Published:** 2022-01-13

**Authors:** Antonios Vitalis, Alena Shantsila, Mark Kay, Rajiv K. Vohra, Gregory Y. H. Lip

**Affiliations:** 1 Liverpool Centre for Cardiovascular Science, University of Liverpool and Liverpool Heart and Chest Hospital, Liverpool, UK; 2 Institute of Cardiovascular Sciences, University of Birmingham, Birmingham, UK; 3 Department of Vascular Surgery, University Hospitals Birmingham NHS Foundation Trust, Queen Elizabeth Hospital Birmingham, Birmingham, UK

**Keywords:** ethnicity, peripheral arterial disease, angioplasty, endovascular

## Abstract

**Purpose:**

Various studies, mainly from North America, report worse outcomes in ethnic minority populations submitted to revascularization for peripheral arterial disease (PAD). Limited nationwide data in relation to ethnicity are available from Europe.

**Objective:**

The objective of the study is to compare the outcomes of femoral angioplasty/stenting procedures among different ethnic groups in England during the 10-year period from 2006 to 2015.

**Materials and Methods:**

The “Hospital Episode Statistics” database has been searched using *International Classification of Diseases, Tenth Revision* (*ICD-10*) codes to identify all cases of femoral angioplasty or stenting from English NHS Hospitals between January 1, 2006, and December 31, 2015. Subsequent mortality, second open or endovascular infrainguinal procedures, and major amputations on the same side within 2 years after the first procedure have been recorded. Patients were broadly categorized according to ethnicity as whites, Asians, and blacks. Chi-square test was used to demonstrate significant differences among ethnic groups and odds ratios (ORs) were calculated using white ethnic group as reference.

**Results:**

A total number of 70 887 femoral endovascular procedures were recorded in patients from the 3 ethnic groups. Two-year mortality in whites, Asians, and blacks was 18.3%, 22.1%, and 19.5% (p<0.001); rates of second endovascular procedure were 12.1%, 13.1%, and 13.5% (p=0.24); rates of open infrainguinal procedure were 5.6%, 4.5%, and 8.0% (p<0.001); and rates of major amputation were 4.8%, 4.1%, and 7.0% (p<0.001), respectively. Mortality was higher in Asians (OR=1.26, 95% confidence interval [CI]=1.10-1.45, p<0.01) compared with whites. On the contrary, blacks underwent more open arterial operations (OR=1.48, 95% CI=1.19-1.83, p<0.01) and more amputations (OR=1.49, 95% CI=1.18-1.87, p<0.01). There were no significant differences in the rates of second endovascular procedures.

**Conclusion:**

Two-year mortality after femoral angioplasty/stenting is higher in Asians, whereas risk of limb loss is higher in blacks compared with whites. Reasons of these ethnic differences in outcomes following femoral endovascular procedures for PAD merit further study.

## Introduction

There is evidence that epidemiology, risk factor profile, and outcomes vary between different ethnic groups. Black ethnicity is associated with higher rates of hypertension,^1–4^ higher prevalence of peripheral arterial disease (PAD),^5^ and higher risk of amputation.^6–8^ On the contrary, Asian ethnicity is associated with higher rates of diabetes,^9–11^ but despite that lower prevalence of PAD^5^ and lower risk of amputation.^7^

Various studies have demonstrated worse outcomes after revascularization procedures in ethnic minority groups. Most of that evidence originates from North America, whereas very limited evidence is available for ethnic minority populations in Europe. Whether these differences can be attributed to different prevalence of cardiovascular risk factors, socioeconomic, or genetic factors remains unclear. Ethnic minorities comprise 14% of population of England with the main ethnic groups being Asians (7.5%) and black/African/Caribbean (3.3%).^12^

We therefore conducted a retrospective study of English hospital statistics to identify possible differences in outcomes of peripheral endovascular procedures in these groups. We hypothesized that differences in outcomes of revascularization procedures observed in the United States would also apply in ethnic minority populations in England.

## Materials and Methods

This is a retrospective study using UK Hospital Episode Statistics (HES). The HES is the administrative data set for the English National Health Service (NHS), which contains information regarding every admission of any patient to English NHS hospitals. The HES data are anonymized by the allocation of a unique identifier to each patient, so individuals can be tracked as their care moves from consultant to consultant on any particular admission, and between hospital admissions. The data set therefore allows long-term follow-up of individual patients with respect to multiple hospital admissions. Advantages of such data sets have been documented in literature as they encompass large populations, are easily available, and are amenable to computerized data extraction.^13^ *International Classification of Diseases, Tenth Revision* (*ICD-10*) codes^14^ (Supplementary Table 1) were used to detect corresponding clinical diagnoses and different treatments. Ethnicity is self-defined by patients on admission, and as reported before, it is recorded in the HES database in 79.4% of hospital admissions.^15^

**Table 1.** Baseline Characteristics in Different Ethnic Groups.

	Whites	Asians	Blacks	p^a^	p^b^
N	73 041	1188	1224		
Mean age (SD)	71.7 (11.4)	67.4 (11.5)	70.6 (12.4)	<0.01	<0.01
Male (%)	62.4	78.6	58.0	<0.01	<0.01
Diabetes (%)	32.7	68.9	59.9	<0.01	<0.01
Hypertension (%)	46.4	53.4	54.3	<0.01	<0.01
Ischemic heart disease (%)	24.2	41.0	21.2	<0.01	0.02
Heart failure (%)	5.3	6.9	8.0	0.01	<0.01
Stroke (%)	0.3	0.8	0.8	<0.01	<0.01
Atrial fibrillation (%)	11.8	6.0	7.6	<0.01	<0.01

a Comparing whites with Asians.

b Comparing whites with blacks.

We searched HES database to detect all femoral angioplasty and/or stenting procedures performed in English NHS Hospitals during the 10-year period between January 1, 2006, and December 31, 2015. Based on recorded ethnicity, patients were categorized in 1 of the 3 main ethnic groups: white, Asian, black/African/Caribbean.^12^ Patients with recorded “Mixed Ethnicity” or missing ethnicity were excluded from the study. Demographic characteristics and previous diagnosis of hypertension, diabetes, heart failure, ischemic heart disease (IHD), stroke, and atrial fibrillation (AF) were recorded using relevant *ICD-10* codes.

**Figure 1.**
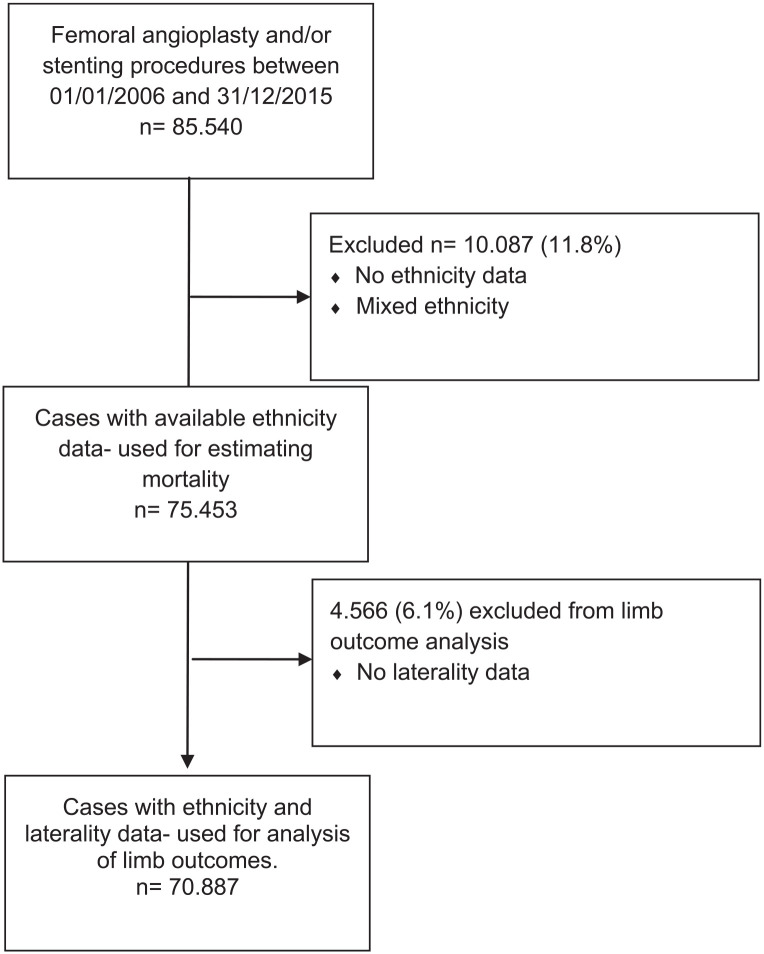
Study flow diagram.

Every patient’s records were studied for a 2-year period after the initial endovascular intervention and the outcomes examined were 30-day and 2-year mortality, reinterventions, and amputations on the same limb. In an attempt to only include subsequent procedures in the same arterial segment, only infrainguinal open or endovascular procedures were recorded. A comparison of baseline characteristics and outcomes was made between patients of different ethnic groups using descriptive statistics. Only procedures with laterality codes, allowing matching the side of the initial procedure with the side of subsequent events, were included in the analysis of limb-related outcomes. For estimation of mortality, the whole data set was used, regardless of laterality codes (Figure 1). Chi-square test was used and p value was calculated to demonstrate significant differences, defined as p<0.05, using MedCalc Version 14.8.1. Odds ratios were calculated using white ethnic group as reference.

## Results

In the time period from 2006 to 2015, a total number of 85 540 patients had their first endovascular procedure in the femoral artery, with 75 453 (88.2%) having ethnicity data as follows: 68 622 (96.8%) white, 1121 (1.6%) Asian, and 1144 (1.6%) black ethnicity. Asian patients were significantly younger, more likely to be male, and had higher prevalence of diabetes and IHD compared with other ethnic groups (Table 1). Black patients had higher prevalence of hypertension and heart failure, whereas whites had higher prevalence of AF. Asians and blacks had equally higher rates of previous stroke compared with whites (Table 2).

**Table 2.** Outcomes of Infrainguinal Endovascular Procedures in Different Ethnic Groups.

	Whites	Asians	Blacks	p^a^	p^b^
Mortality					
n	73 041	1188	1224		
30-day mortality (%)	2.0	3.5	2.0	<0.01	0.96
2-year mortality (%)	18.3	22.1	19.5	<0.01	0.30
Limb outcomes					
n	68 622	1121	1144		
Open operation (%)	5.6	4.5	8.0	0.15	<0.01
Second endovascular procedure (%)	12.1	13.1	13.5	0.34	0.18
Major amputation (%)	4.8	4.1	7.0	0.31	<0.01

Only procedures with laterality codes used for limb outcomes.

a Comparing whites with Asians.

b Comparing whites with blacks.

Overall, 30-day and 2-year mortality in this population were 2% and 18.4% with Asians having significantly higher 30-day (OR=1.73, 95% CI=1.26-2.37, p<0.01) and 2-year (OR=1.26, 95% CI=1.10-1.45, p<0.01) mortality compared with whites. There was no significant difference in mortality between blacks and whites (Table 3).

**Table 3.** Studies Comparing Revascularization Outcomes in Different Ethnic Groups.

Author	Year	Study type	Data source	Years studied	Population	Intervention	Mortality	Limb outcomes
Singh et al^16^	2008	Retrospective	National Surgical Quality Improvement Program database (USA)	1995-2003	W: 10 602 B: 2578	All infrainguinal bypass		30-day graft failure: W=4.5%, B=6.7% OR=1.40; 95% CI=1.30-1.50, p<0.01
Robinson et al^17^	2009	Retrospective	Single-center database	1985-2007	W: 1408 B: 181	Infrainguinal bypass with autologous vein	30-day: W=2.3%, B=2.8% (p=0.47) 5-year survival: W=61%, B=58%	30-day graft failure: W=5%, B=11% 5-year primary patency: W=69%, B=58% (p<0.01) 5-year limb salvage: W=91%, B=84% (p<0.01)
Nguyen et al^18^	2009	Retrospective	PREVENT III multicentre trial data set including patients with critical ischemia (North America)		B: 249 O: 1155	Infrainguinal bypass with autologous vein	30-day: Overall 2.7%, no significant difference 1-year: Overall 16%, no significant difference	30-day graft failure: higher in black men (OR=2.99; 95% CI=1.74-6.07, p<0.01) 1-year primary patency: no difference 1-year amputation: overall 16%, higher in blacks (HR=2.00; 95% CI=1.20-3.33, p<0.01)^a^
Tiwari et al^19^	2011	Retrospective	Single-center database (Kings College Hospital, London)	2004-2009	W: 86 B: 39	Distal bypass for critical leg ischemia	30-day: W=2.3%, B=2.6%	1-year primary patency: W=44.6%, B=51.3%, p=0.46 No difference in primary-assisted, secondary patency and amputation-free survival
Chong et al^20^	2011	Retrospective	Single-center medical records (California)	1994-2007	280 patients 374 procedures W: 60% B: 12% A: 5%	Endovascular interventions on femoral and popliteal arteries		Mean FU 3.6 years- primary failure Overall: no difference, p=0.12 Stenting procedures: no difference, p=0.48 Angioplasty procedures: W=60%, B=39%, A=29% p<0.01
Selvarajah et al^21^	2014	Retrospective	National Surgical Quality Improvement Program database (USA)	2005-2011	W: 12 536 B: 2940	All infrainguinal bypass	30-day: W=2.3%, B=2.9% (p=0.12)	30-day graft failure: W=15.4%, B=16.6% OR=1.26; 95% CI=1.05-1.51, p=0.01^a^
Loja et al^22^	2015	Retrospective	Patient Discharge Data from California’s Office of Statewide Health Planning and Development	2005-2009	W: 17 433 B: 1163	All peripheral endovascular interventions	1-year: W=9.8%, B=12.5%	30-day major amputation: W=1.7%, B=6.2% OR=1.99; 95% CI=1.56-2.55; p<0.01^a^ 1-year major amputation: W=4.1%, B=13.8% OR=1.85; 95% CI=1.54-2.12, p<0.01^a^ 1-year reintervention: W=32.9%, B=36.6% OR=1.17; 95% CI=1.06-1.30, p<0.01^a^
Rivero et al^23^	2016	Retrospective	Single-center database (New York)	2003-2012	W: 925 B: 137 (all male)	Any revascularization (open, endovascular, hybrid)	30-day open: W=1.9%, B=4.8% (p=0.42) 30-day endovascular: W=1.4%, B=0% (p=0.28)	5-year limb salvage Open: No difference (p=0.20) Endovascular: W=84% B=69% (p=0.03) Hybrid: No difference (p=0.25) No difference in primary patency rates
Yang et al^24^	2019	Retrospective	National Surgical Quality Improvement Program database (USA)	2013	W: 1732 B: 288	All infrainguinal bypass	30-day: W=2%, B=2% (p=0.9)	30-day limb loss: W=3%, B=8%, OR=2.8; 95% CI=1.76-4.56, p<0.01^a^

W = white; B = black; A = Asian; O = Other; OR = odds ratio; CI = confidence interval; HR = hazard ratio; FU = follow-up.

a Multivariate adjustment.

Limb-related outcomes with known laterality were available in 70 887 patients. There was no significant difference among ethnic minority groups in the rates of second endovascular procedure in the 2-year period. However, black patients were more likely to need open revascularization (OR=1.48, 95% CI=1.19-1.83, p<0.01) and were at higher risk of major amputation (OR=1.49, 95% CI=1.18-1.87, p<0.01) compared with whites. There was no significant difference in limb outcomes between Asians and whites (Table 3).

## Discussion

The principal finding of this study is the higher risk of limb loss for black patients who underwent infrainguinal endovascular interventions. In addition, black patients were more likely to need a subsequent open revascularization procedure after the initial femoral angioplasty. Asian patients in our study had similar rates of limb loss and reinterventions to white patients, despite their different risk factor profile and higher prevalence of diabetes.

Two previous studies investigating the outcome of endovascular procedures reach similar conclusions. Loja et al^22^ report higher reintervention rates and higher amputation rates in blacks, 1 month and 1 year after endovascular procedures for PAD. Rivero et al^23^ also report worse limb salvage rates 5 years after endovascular procedures in black patients compared with whites (69% vs 84%) despite patency rates being similar for both ethnic groups. Another study on femoral and popliteal endovascular interventions by Chong et al^20^ reports similar patency rates among white, black, and Asian patients. The above evidence comes from the United States and our study is the first to report similar observations within a different population and health care system. We believe this is a meaningful addition to the existing data.

In our study ethnic groups present with different characteristics and risk factor profiles. Asians are younger, are more likely to be male, and present with higher prevalence of diabetes, followed by blacks and whites. Blacks present with higher rates of hypertension compared with the other ethnic groups and whites have higher rates of AF. These differences in medical history are present in previous studies on patients receiving peripheral angioplasties.^20,22,23^ These findings are also in accordance with previous reports from general population studies^2–4,25^ as well as studies on patients with atherosclerotic disease.^26^ Several studies have performed regression analysis to eliminate the effect of these factors on outcomes and concluded that black ethnicity remains an independent risk factor for limb loss and worse patency rates after open^21,24^ or endovascular interventions.^22^

Another important finding is the higher mortality in patients of Asian ethnicity, despite their younger age. This may reflect the higher prevalence of IHD and diabetes in this group. In fact, patients of Asian descent present with different distribution of atherosclerotic disease and have been reported to have higher rates of coronary artery disease and lower rates and lower severity of PAD.^5,9,27^ On the contrary, black and white patients present with similar mortality rates, which is consistent in all studies comparing mortality rates after open^17–19,21,23^ or endovascular^22,23^ procedures.

A search of literature was conducted to correlate our results with previously published studies. We enquired the Medline database for studies comparing outcomes of revascularization procedures between white, black, and Asian ethnic groups. Nine studies comparing outcomes of vascular procedures between ethnic groups under investigation have been identified, 8 from North America and 1 from the United Kingdom. All studies were retrospective; 2 studies compare peripheral endovascular procedures, 6 open bypass operations, and 1 both. All studies include a comparison between black and white ethnicity but only 1 study investigates Asian ethnicity. Black patients presented with higher risk of early graft failure after infrainguinal bypass procedure in 5 studies and with lower limb salvage rates after an infrainguinal bypass procedure (3 studies) or a peripheral endovascular procedure (2 studies). On the contrary, 2 studies did not demonstrate any difference in limb salvage rates. A summary of the studies and main findings is included in Table 3.

This article adds on the existing evidence about racial disparities on outcomes of revascularization procedures. Further research on potential genetic, environmental, or socioeconomic factors that generate these disparities is necessary and every effort should be made to eliminate them.

### Strengths and Limitations

Main strength of this study is the large number of patients and procedures, as it attempted to include all femoral endovascular procedures that took place in England during the studied period. However, it has certain limitations. The large number of cases could generate statistically significant differences that may lack clinical significance. It is of retrospective nature and clinical diagnosis is based on a coding system, which lacks the accuracy of a clinical trial. In addition, ethnic minority groups are very underrepresented in this study with a significant proportion of cases (11.8%) having been excluded due to missing ethnicity data or “mixed ethnicity,” and, in addition, detailed socioeconomic status was not available. We used the broad categorization of ethnic groups as they appear in census data and in the UK Hospital Statistics coding, which are used in HES data. Our data originate from 1 healthcare system and should not be generalized to other systems without some caution. There have been no data on the clinical severity of the disease or the presence of critical ischemia at the time of intervention and no information about the anatomic distribution or the complexity of the treated lesion or use of drug eluting devices. Most importantly, we were not able to perform a full multivariate regression to account for the possible effect of confounders, including the kidney function, due to our level of access to data.

In conclusion, 2-year mortality after femoral angioplasty/stenting is higher in Asians, whereas risk of limb loss is higher in blacks compared with whites. Reasons of these ethnic differences in outcomes following femoral endovascular procedures for PAD merit further study.

## Supplemental Material

sj-docx-1-jet-10.1177_15266028211070967 – Supplemental material for Outcome of Femoral Angioplasty/Stenting Procedures in Different Ethnic Groups in England: A Retrospective Analysis of Hospital Episode Statistics and Review of Literature Click here for additional data file. Supplemental material, sj-docx-1-jet-10.1177_15266028211070967 for Outcome of Femoral Angioplasty/Stenting Procedures in Different Ethnic Groups in England: A Retrospective Analysis of Hospital Episode Statistics and Review of Literature by Antonios Vitalis, Alena Shantsila, Mark Kay, Rajiv K. Vohra and Gregory Y. H. Lip in Journal of Endovascular Therapy
