# Supplementary material for: Outcome of Femoral Angioplasty/Stenting Procedures in Different Ethnic Groups in England: A Retrospective Analysis of Hospital Episode Statistics and Review of Literature
Source: J Endovasc Ther. 2022 Jan 13;30(1):132–9. doi: 10.1177/15266028211070967 (PMC9896409; doi:10.1177/15266028211070967)
Supplement: sj-docx-1-jet-10.1177_15266028211070967 – Supplemental material for Outcome of Femoral Angioplasty/Stenting Procedures in Different Ethnic Groups in England: A Retrospective Analysis of Hospital Episode Statistics and Review of Literature [file sj-docx-1-jet-10.1177_15266028211070967.docx]

Supplementary Table 1. Codes used to identify infrainguinal endovascular procedures and subsequent outcomes.

| ICD-10 code | Intervention |
| --- | --- |
| Endovascular procedure | |
| L63.1 | Percutaneous transluminal angioplasty of femoral artery |
| L63.5 | Percutaneous transluminal insertion of stent into femoral artery |
| Open reintervention | |
| L58* | Other emergency bypass of femoral artery |
| L59* | Other bypass of femoral artery |
| L60* | Reconstruction of femoral artery |
| L62.2 | Open embolectomy of femoral artery |
| L65.3 | Revision of reconstruction involving femoral artery |
| Endovascular reintervention | |
| L63* | Transluminal operations on femoral artery |
| Amputation | |
| X09.3 | Amputation of leg above knee |
| X09.4 | Amputation of leg through knee |
| X09.5 | Amputation of leg below knee |

(*= including subcategories) **Index Procedure**

L63.1 Percutaneous transluminal angioplasty of femoral artery

L63.5 Percutaneous transluminal insertion of stent into femoral artery

**Subsequent open procedure**

L58 Other emergency bypass of femoral artery + subcategories

L59 Other bypass of femoral artery + subcategories

L60 Reconstruction of femoral artery + subcategories

L62.2 Open embolectomy of femoral artery

L65.3 Revision of reconstruction involving femoral artery

**Subsequent endovascular procedure**

L63 Transluminal operations on femoral artery + subcategories

**Major Amputation**

X09.3Amputation of leg above knee

X09.4Amputation of leg through knee

X09.5Amputation of leg below knee

**Diabetes**

E10 Type 1 diabetes mellitus

E11 Type 2 diabetes mellitus

E14 Unspecified diabetes mellitus

**Hypertension**

I10 Essential (primary) hypertension

I15 Secondary hypertension

I15.0 Renovascular hypertension

I15.1 Hypertension secondary to other renal disorders

I15.2 Hypertension secondary to endocrine disorders

I15.8 Other secondary hypertension

I15.9 Secondary hypertension, unspecified

**Ischaemic Heart Disease**

I20 Angina pectoris

I20.0 Unstable angina

I20.1 Angina pectoris with documented spasm

I20.8 Other forms of angina pectoris

I20.9 Angina pectoris, unspecified

I21 Acute myocardial infarction

I21.0 Acute transmural myocardial infarction of anterior wall

I21.1 Acute transmural myocardial infarction of inferior wall

I21.2 Acute transmural myocardial infarction of other sites

I21.3 Acute transmural myocardial infarction of unspecified site

I21.4 Acute subendocardial myocardial infarction

I21.9 Acute myocardial infarction, unspecified

I22 Subsequent myocardial infarction

I22.0 Subsequent myocardial infarction of anterior wall

I22.1 Subsequent myocardial infarction of inferior wall

I22.8 Subsequent myocardial infarction of other sites

I22.9 Subsequent myocardial infarction of unspecified site

I24 Other acute ischaemic heart diseases

I24.0 Coronary thrombosis not resulting in myocardial infarction

I24.1 Dressler syndrome

I24.8 Other forms of acute ischaemic heart disease

I24.9 Acute ischaemic heart disease, unspecified

I25 Chronic ischaemic heart disease

I25.0 Atherosclerotic cardiovascular disease, so described

I25.1 Atherosclerotic heart disease

I25.2 Old myocardial infarction

I25.3 Aneurysm of heart

I25.4 Coronary artery aneurysm and dissection

I25.5 Ischaemic cardiomyopathy

I25.6 Silent myocardial ischaemia

I25.8 Other forms of chronic ischaemic heart disease

I25.9 Chronic ischaemic heart disease, unspecified

**Heart Failure**

I50 Heart failure

I50.0 Congestive heart failure

I50.1 Left ventricular failure

I50.9 Heart failure, unspecified

**Stroke**

I63 Cerebral infarction + subcategories

Ι60 Subarachnoid haemorrhage + subcategories

I61 Intracerebral haemorrhage + subcategories

I62 Other nontraumatic intracranial haemorrhage

**Atrial Fibrillation**

I48 Atrial fibrillation and flutter

I48.0 Paroxysmal atrial fibrillation

I48.1 Persistent atrial fibrillation

I48.2 Chronic atrial fibrillation
